# Oncological and physiological impact of thoracic duct resection in esophageal cancer

**DOI:** 10.1093/dote/doad015

**Published:** 2023-03-22

**Authors:** Satoru Matsuda, Masashi Takeuchi, Hirofumi Kawakubo, Hiroya Takeuchi, Yuko Kitagawa

**Affiliations:** Department of Surgery, Keio University School of Medicine, Tokyo, Japan; Department of Surgery, Keio University School of Medicine, Tokyo, Japan; Department of Surgery, Keio University School of Medicine, Tokyo, Japan; Department of Surgery, Hamamatsu University School of Medicine, Hamamatsu, Japan; Department of Surgery, Keio University School of Medicine, Tokyo, Japan

## Abstract

Despite advances in multidisciplinary treatment, esophagectomy remains the main curative treatment for esophageal cancer. The advantages and disadvantages of thoracic duct (TD) resection have been controversial for decades. We have herein reviewed relevant published literature regarding ‘thoracic duct,’ ‘esophageal cancer,’ and ‘esophagectomy’ describing the anatomy and function of the TD, and incidence of thoracic duct lymph nodes (TDLN) and TDLN metastases, as well as the oncological and physiological effects of TD resection. The presence of lymph nodes around the TD, referred to as TDLN, has been reported previously. The delineation of TDLNs is clearly defined by a thin fascial structure covering the TD and the surrounding adipose tissue. Previous studies have examined the number of TDLNs and the percentage of patients with TDLN metastasis and revealed that each patient had approximately two TDLNs. The percentage of patients with TDLN metastasis was reported to be 6–15%. Several studies have been conducted to compare the survival after TD resection with that after TD preservation. However, no consensus has been reached because all studies were retrospective, precluding firm conclusions. Although the issue of whether the risk of postoperative complications is affected by TD resection is still unclear, resecting the TD has been shown to have a long-term impact on nutritional status after surgery. In summary, TDLNs are quite common and present in most patients, while metastasis in the TDLNs occurs in a minority. However, the oncological value of TD resection in esophageal cancer surgery remains controversial due to varying findings and methodological limitations of previous comparative studies. Considering the potential but unproven oncological benefits and possible physiological drawbacks of TD resection, including postoperative fluid retention and disadvantages in the long-term nutritional outcome, clinical stage, and nutritional status should be considered before deciding whether to perform TD resection or not.

## INTRODUCTION

Esophageal cancer is one of the most fatal diseases because it spreads rapidly even in its early stages.^1^^,^^2^ Despite advances in multidisciplinary treatment,^3^^,^^4^ esophagectomy remains the main curative treatment for esophageal cancer (both esophageal squamous cell carcinoma [ESCC] and adenocarcinoma [AC]). Extensive lymph node (LN) dissection has been a key component of surgical resection because achieving R0 resection is critical for long-term survival after esophagectomy. Extended lymphadenectomy, also known as three-field LN dissection, can improve survival and specifically developed for treating ESCC, which is the most commonly occurring type in Asian countries.^5^^,^^6^

As the thoracic duct (TD) is one of the key anatomical components resected in esophagectomy, the advantages and disadvantages of its resection have been controversial for decades. Although TD resection may increase the likelihood of disease cure, there are potential drawbacks due to the physiological function of the TD. We herein reviewed relevant published literature to study the clinical significance of TD resection in esophageal cancer surgery from multiple perspectives, including prognostic impact, postoperative complications, and short- and long-term effects on patients after surgery.

We herein reviewed relevant published literature regarding ‘thoracic duct,’ ‘esophageal cancer,’ and ‘esophagectomy’ to evaluate the anatomy, function of the TD, and thoracic duct lymph node (TDLN) as well as the oncological and physiological effects of TD resection.

## ANATOMY AND FUNCTION OF TD AND TDLN

The main lymphatic root, the TD, arises from the chyle cistern and ascends along the thoracic descending aorta. In the upper mediastinum, the TD is located on the posterolateral side of the esophagus and eventually flows into a left venous angle. The anatomical patterns of the TD were classified into nine types in the early 20th century.^7^ While assessing the clinical importance of TD resection in esophageal cancer surgery, the presence of LNs with metastatic tumor sites must be considered. Udagawa *et al.* reported the presence of LNs around the TD, particularly in what has been described as TDLN. A thin fascial structure covering the TD and the surrounding adipose tissue represents the boundaries of the TDLN.^8^ Recently, Tokairin *et al.* used microanatomy in a cadaver study to visualize that a membranous fascia composed of dense connective tissue surrounds the TD.^9^

## ONCOLOGICAL IMPACT OF TD RESECTION IN ESOPHAGEAL CANCER SURGERY

### TDLN metastasis and its prognostic impact

The oncological impact of TD resection in esophageal cancer surgery has long been debated. When considering TD resection for esophageal cancer, it is expected that TD resection combined with TDLN removal will improve the local control and radicality. Udagawa *et al.*^8^ examined 778 patients who had undergone transthoracic esophagectomy with TD resection. The TDLN metastatic incidence was 2.2% in pT1b/pT2 and 10.0% in pT3/pT4. As TDLNs exist around the TD, they concluded that TD resection with dissection should be performed regularly. These findings were validated in a follow-up study conducted by the same institution with a larger cohort.^10^ In Europe, where adenocarcinoma is common and Ivor Lewis esophagectomy is routinely performed, Defize *et al.*^11^ conducted a multi-institutional observational study and revealed the presence of TDLNs in approximately 50% of the patients, with a metastatic rate of 15% (Table 1).

**Table 1:** Presence of TDLN and metastasis

Author (year)	*n*	Number of TDLNs per patient	Percentage of patients with TDLN metastasis		
			All	pT1–2	pT3–4
Udagawa (2014), Ohkura (2022)	1,211	1.7 ± 2.1	6.6%	2%	12%
Matsuda (2016, 2020)	232	2.03 ± 2.47	7%	3%	17%
Defize (2022)	117	2	15%	2%	18%

**Fig. 1:**
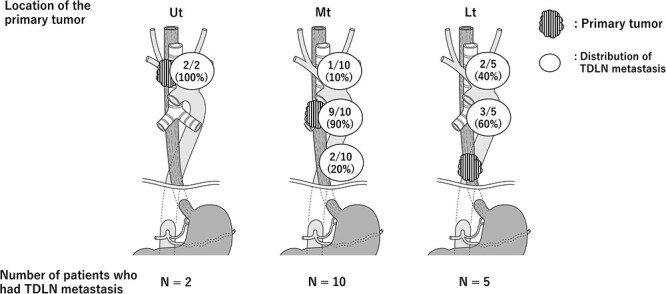
Distribution of TDLN based on primary tumor location.

Subsequently, we investigated the presence and metastatic rate of TDLN in esophageal cancer surgery.^12^^,^^13^ As shown in Table 1, the average number of TDLNs, including nonmetastatic nodes, was comparable across studies; this was confirmed in a cadaver study revealing the presence of TDLNs in six of seven cadavers (86%).^14^ In our study, TDLN metastasis was observed in 7% of patients with surgically resectable ESCC, similar to the finding of a previous study.^8^^,^^10^ Next, we confirmed that TDLN metastasis occurred in the advanced stages of the disease and that patients with TDLN metastasis had multiple metastatic locations. None of the patients in the current study had solitary LN metastasis in TDLN without non-TDLN metastasis, suggesting that TDLN does not receive direct lymphatic flow from the primary tumor. The location of TDLN metastasis revealed that TDLN-Ut (upper thoracic)/Mt (mid-thoracic)/Lt (lower thoracic) metastasis occurred in 29%/71%/12% of cases, respectively, indicating that TDLN metastasis occurred frequently in the mid-thoracic esophagus. Regarding the anatomical relationship between TDLN metastasis and other metastatic lesions, we demonstrated that the primary tumor or LN metastasis in LNs other than TDLN was located at the same or caudal level as the TDLN metastasis. Figure 1 summarizes the distribution of TDLN metastasis based on primary tumor location, demonstrating that TDLN metastasis tends to occur at the same level as the primary tumor or cranial it. This finding was confirmed in a follow-up study conducted by the same institution using a larger cohort,^12^^,^^13^ which revealed that the lymphatic route along the TD could extend from caudal to cranial levels. In terms of prognosis, recurrence-free survival (RFS) and overall survival (OS) were significantly worse in patients with TDLN metastasis, which was shown to be an independent prognostic factor in the multivariate analysis. In particular, TDLN metastasis had a negative prognostic impact in TDLN-Mt/Lt. Overall, the findings suggest that TDLN metastasis is a strong negative prognostic factor.

### Effect on survival of TDLN dissection and comparison between TD resection and preservation

The presence of TDLNs does not imply that TDLN resection improves survival. Thus, its impact on survival should be evaluated based on the prognosis of patients with TDLN resection who have TDLN metastasis. According to a previous study, the prognosis of patients with TDLN metastasis was not worse than that of patients with non-TDLN metastasis.^8^ In our previous study, the RFS and OS of patients with TDLN metastasis were similar to those with positive metastasis in extra-regional LNs, such as supraclavicular LN.^13^ Therefore, given that supraclavicular LN dissection has moderate efficacy with regard to survival for ESCC located in the upper mid-thoracic esophagus, TDLN dissection may be beneficial.^15^ The efficacy index (EI) is calculated by multiplying the frequency (%) of metastases for each LN station by the 5-year survival rate (%) of patients with metastases.^16^ Ohkura *et al.*^10^ compared the EI of TDLN and non-TDLN lymph node stations and revealed that the EI of TDLN was comparable to or higher than that of other regional LNs.

Table 2 summarizes the comparison between the TD-preserved and -resected groups for evaluating the overall advantages and disadvantages of TD resection. Tanaka *et al.*^17^ examined 2,269 patients with ESCC who had undergone transthoracic esophagectomy. The OS and disease-free survival in the TD-preserved and -resected groups were compared. Propensity score matching revealed that the OS was significantly better in the TD-resected group than in the TD-preserved group, particularly in advanced stages. As hematogenous recurrence was lower in the TD-resected group, they concluded that TD resection may help improve prognosis in patients with advanced diseases.

**Table 2:** Comparison of the preserved and resected thoracic duct groups

	Treatment	*n*	Postoperative complication	Number of retrieved lymph nodes	Survival
Tanaka (2021)	TD-preserved TD-resected	642 642 ^*^PM	NA	Higher in TD-resected group	OS improvement in stages III/IV
Oshikiri (2021)	TD-preserved TD-resected	1638 1638 ^*^PM	NS	Higher in TD-resected group	NS
Oshikiri (2019)	TD-preserved TD-resected	122 122 ^*^PM	Higher incidence of chylothorax and left recurrent laryngeal nerve palsy in TD-resected group	Higher in TD-resected group	NS
Matsuda (2020)	TMIE E-TMIE	44 191	NS	Higher in TD E-TMIE group	RFS improvement in cT1N0M0
Yoshida (2019)	TD-preserved TD-resected	342 145	Higher incidence of pulmonary complication in TD-resected group	Higher in TD-resected group	NS

NA, not assessed; NA, not statistically significant; PM, propensity score matching; RFS, recurrence-free survival; TD, thoracic duct; E-TMIE, transthoracic minimally invasive esophagectomy with extended lymph node dissection and thoracic duct resection; TMIE, transthoracic minimally invasive esophagectomy.

We also previously reported the clinical significance of transthoracic esophagectomy with extended LN dissection (E-TMIE).^18^ The number of retrieved mediastinal LNs was higher in patients who underwent E-TMIE than in those who underwent TMIE. RFS was also better in the E-TMIE group, particularly in patients with cT1N0M0 disease. We concluded that radical LN dissection, including TD resection, may improve local control and survival because solitary LN recurrence in the mediastinum was not observed in the E-TMIE group. When TD is resected during esophagectomy, the adipose tissue surrounding the esophagus, which may contain a tumor, can be removed concurrently, potentially increasing the radicality of surgical resection. Moreover, as previously reported, TD resection is associated with an increase in the number of LNs not only in TDLNs but also in LNs surrounding the recurrent laryngeal nerves due to extensive LN dissection. These multiple roles of TD resection support our previous findings that extensive LN dissection combined with TD resection improves survival, particularly in cStage I ESCC, where surgical resection without perioperative therapy is the standard of care.

In contrast, a recent retrospective study conducted with a larger cohort did not support the survival benefit of TDLN. Oshikiri *et al.*^19^ examined 12,237 patients from the Japan Esophageal Society’s comprehensive registry data. Using propensity score matching, they compared TD-preserved and -resected cohorts. Although TD resection was significantly associated with an increase in the number of retrieved LNs, there was no survival benefit of TD resection.

Overall, although the presence of TDLNs has been validated by several studies and the efficacy of TDLN dissection has been previously suggested, the survival benefit of TD resection remains controversial. A significant limitation is that most comparative studies were conducted retrospectively. Considering that TD preservation is currently a standard surgical procedure in >50% of institutions, the TD-resected group is expected to have more advanced-stage disease despite the addition of propensity score matching or multivariate analysis. Prospective interventional studies are warranted for a fair comparison of TD resection and preservation.

## PERIOPERATIVE RISKS AND PHYSIOLOGICAL EFFECTS OF TD RESECTION

Because TD transports large amounts of chyle from the abdomen to systemic circulation, its removal could result in various physiological effects. TD ligation has been linked to retroperitoneal fluid retention and an increase in intravenous volume requirement after surgery.^20^ Furthermore, because lymphatic drainage from the small intestine flows into the chyle cistern, either ligation or resection would impair nutrient absorption. Indeed, Aiko *et al.*^21^ reported that TD resection altered fluid balance and decreased the clinical benefit of enteral feeding following esophagectomy. Furthermore, TD ligation had a negative effect on the liver in a canine model of peritonitis, which was induced by high exposure to endotoxin in the liver.^22^

Because esophagectomy is one of the most invasive gastrointestinal surgeries and is associated with high morbidity and mortality, the main concern is increased postoperative risk after TD resection.^23^ In our previous study demonstrating that E-TMIE improved the long-term outcomes, particularly in the early stage, postoperative complications did not increase,^18^ which was consistent with other studies that showed the oncological benefit of TD resection with no increase in postoperative complications.^17^ While a previous study reported that intraoperative TD mass ligation decreases the risk of chylothorax, ligating the TD is a procedure that is completely different from TD resection.^24^ In fact, postoperative complications after TD resection did not increase in another study focusing primarily on Ivor Lewis esophagectomy for AC.^11^ However, a previous study reported that TD resection increased the risk of postoperative pneumonia following esophagectomy.^25^ The operative time was significantly longer in the TD-resected group, with frequent pulmonary complications. Another study reported that TD resection was associated with an increased risk of chylothorax and recurrent laryngeal nerve palsy.^26^ Overall, the short-term effects of TD resection remain controversial.

The loss of TD function may impact the nutritional status following esophagectomy. Fujisawa *et al.* compared total body weight, body mass index, and fat mass between the TD-resected and TD-preserved groups after esophagectomy.^27^ The magnitude of reduction in these factors was greater in the TD-resected group at 12 months after surgery, but TD resection had no effect on the skeletal muscle. We recently investigated the impact of TD resection on mid- to long-term body composition.^28^ To rule out the effect of residual and recurrent tumors after surgery, we limited this study to patients who had no recurrence at 3 years after surgery. At 1 and 3 years after surgery, we found that although muscle mass loss was comparable between the groups, adipose tissue loss was significantly greater in the TD-resected group than in the TD-preserved group. The difference was notably smaller at approximately 5 years after surgery. Thus, we conclude that TD resection in patients with esophageal cancer may be acceptable with no long-term effects on body composition. Table 3 summarizes representative studies investigating the physiological effects of TD resection.

**Table 3:** Physiological effects of thoracic duct resection

	Treatment	*n*	Nutritional status
Nishimura (2022)	TD-preserved TD-resected	61 156	On POY 1 and 3, adipose tissue loss was significantly higher in the TD-resected group.
Fujisawa (2021)	TD-preserved TD-resected	51 123	On POY 1, total body weight, body mass index, and fat mass decreased in the TD-resected group.
Aiko (2003)	TD-preserved + EN TD-preserved + PN TD-ligated + EN TD-ligated + PN	13 12 7 7	In TD-ligated groups, EN caused increase in lymphocyte count with no decrease in C-reactive protein level.

EN, enteral nutrition; PN, parenteral nutrition; POY, postoperative year; TD, thoracic duct.

## CONCLUSION AND FUTURE PROSPECTS

When combined with extensive LN dissection, an oncological benefit of TD resection may be achieved in cT1NM0 esophageal cancer. At present, there is limited evidence that the TD should be routinely resected to improve long-term outcomes in advanced-stage diseases. Furthermore, TD resection is not recommended when patients have preoperative comorbidities due to concerns regarding nutritional disadvantages 1–3 years after surgery.

In the future, the prognostic impact of TD resection in esophageal cancer surgery should be evaluated in a randomized control trial with postoperative survival as the primary endpoint. The physiological effects of TD resection on body composition and quality of life (QOL) must be validated. Furthermore, based on prospective interventional studies, it would be beneficial to determine how we can maintain nutritional status and QOL even after TD resection.

## References

[1] Akutsu Y, Kato K, Igaki H et al. The prevalence of overall and initial lymph node metastases in clinical T1N0 thoracic esophageal cancer: from the results of JCOG0502, a prospective multicenter study. Ann Surg 2016; 264(6): 1009–15. 27420375 10.1097/SLA.0000000000001557

[2] Takeuchi H, Fujii H, Ando N et al. Validation study of radio-guided sentinel lymph node navigation in esophageal cancer. Ann Surg 2009; 249(5): 757–63. 19387329 10.1097/SLA.0b013e3181a38e89

[3] Watanabe M, Otake R, Kozuki R et al. Recent progress in multidisciplinary treatment for patients with esophageal cancer. Surg Today 2020; 50(1): 12–20. 31535225 10.1007/s00595-019-01878-7 PMC6952324

[4] Matsuda S, Takeuchi H, Kawakubo H et al. Current advancement in multidisciplinary treatment for resectable cstage II/III esophageal squamous cell carcinoma in Japan. Ann Thorac Cardiovasc Surg 2016; 22(5): 275–83. 27384595 10.5761/atcs.ra.16-00111 PMC5088392

[5] Akiyama H, Tsurumaru M, Udagawa H et al. Radical lymph node dissection for cancer of the thoracic esophagus. Ann Surg 1994; 220(3): 364–72 discussion 372-3. 8092902 10.1097/00000658-199409000-00012 PMC1234394

[6] Ando N, Ozawa S, Kitagawa Y et al. Improvement in the results of surgical treatment of advanced squamous esophageal carcinoma during 15 consecutive years. Ann Surg 2000; 232(2): 225–32. 10903602 10.1097/00000658-200008000-00013 PMC1421135

[7] Riddell A M, Davies D C, Allum W H et al. High-resolution MRI in evaluation of the surgical anatomy of the esophagus and posterior mediastinum. AJR Am J Roentgenol 2007; 188(1): W37–43. 17179325 10.2214/AJR.05.1795

[8] Udagawa H, Ueno M, Shinohara H et al. Should lymph nodes along the thoracic duct be dissected routinely in radical esophagectomy? Esophagus 2014; 11(3): 204–10.

[9] Tokairin Y, Nakajima Y, Kawada K et al. Histological study of the thin membranous structure made of dense connective tissue around the esophagus in the upper mediastinum. Esophagus 2018; 15(4): 272–80. 29948479 10.1007/s10388-018-0625-9

[10] Ohkura Y, Ueno M, Iizuka T et al. Effectiveness of lymphadenectomy along the thoracic duct for radical esophagectomy. Esophagus 2022; 19(1): 85–94. 34333712 10.1007/s10388-021-00868-4

[11] Defize I L, Gorgels S M C, Mazza E et al. The presence of metastatic thoracic duct lymph nodes in western esophageal cancer patients: a multinational observational study. Ann Thorac Surg 2022; 113(2): 429–35. 33676903 10.1016/j.athoracsur.2021.02.041

[12] Matsuda S, Takeuchi H, Kawakubo H et al. Clinical outcome of transthoracic esophagectomy with thoracic duct resection: number of dissected lymph node and distribution of lymph node metastasis around the thoracic duct. Medicine (Baltimore) 2016; 95(24): e3839. 27310961 10.1097/MD.0000000000003839 PMC4998447

[13] Matsuda S, Kawakubo H, Takeuchi H et al. Prognostic impact of thoracic duct lymph node metastasis in esophageal squamous cell carcinoma. Ann Gastroenterol Surg 2021; 5(3): 321–30. 34095722 10.1002/ags3.12432 PMC8164460

[14] Schurink B, Defize I L, Mazza E et al. Two-field lymphadenectomy during esophagectomy: the presence of thoracic duct lymph nodes. Ann Thorac Surg 2018; 106(2): 435–9. 29580778 10.1016/j.athoracsur.2018.02.047

[15] Tachimori Y, Ozawa S, Numasaki H et al. Efficacy of lymph node dissection by node zones according to tumor location for esophageal squamous cell carcinoma. Esophagus 2016; 13: 1–7. 26752982 10.1007/s10388-015-0515-3 PMC4698372

[16] Udagawa H, Ueno M, Shinohara H et al. The importance of grouping of lymph node stations and rationale of three-field lymphoadenectomy for thoracic esophageal cancer. J Surg Oncol 2012; 106(6): 742–7. 22504922 10.1002/jso.23122

[17] Tanaka K, Yamasaki M, Sugimura K et al. Thoracic duct resection has a favorable impact on prognosis by preventing hematogenous spread of esophageal cancer cells: a multi-institutional analysis of 2269 patients. Ann Surg Oncol 2021; 28(8): 4402–10. 33861403 10.1245/s10434-021-09962-4

[18] Matsuda S, Kawakubo H, Takeuchi H et al. Minimally invasive oesophagectomy with extended lymph node dissection and thoracic duct resection for early-stage oesophageal squamous cell carcinoma. Br J Surg 2020; 107(6): 705–11. 32077101 10.1002/bjs.11487

[19] Oshikiri T, Numasaki H, Oguma J et al. Prognosis of patients with esophageal carcinoma following routine thoracic duct resection: a propensity-matched analysis of 12,237 patients based on the comprehensive registry of esophageal cancer in Japan. Ann Surg 2021. 10.1097/SLA.0000000000005340 34913902

[20] Imamura M, Shimada Y, Kanda T et al. Hemodynamic changes after resection of thoracic duct for en bloc resection of esophageal cancer. Surg Today 1992; 22(3): 226–32. 1392326 10.1007/BF00308827

[21] Aiko S, Yoshizumi Y, Matsuyama T et al. Influences of thoracic duct blockage on early enteral nutrition for patients who underwent esophageal cancer surgery. Jpn J Thorac Cardiovasc Surg 2003; 51(7): 263–71. 12892455 10.1007/BF02719376

[22] Guler O, Ugras S, Aydin M et al. The effect of lymphatic blockage on the amount of endotoxin in portal circulation, nitric oxide synthesis, and the liver in dogs with peritonitis. Surg Today 1999; 29(8): 735–40. 10483748 10.1007/BF02482318

[23] Marubashi S, Takahashi A, Kakeji Y et al. Surgical outcomes in gastroenterological surgery in Japan: report of the National Clinical Database 2011-2019. Ann Gastroenterol Surg 2021; 5(5): 639–58. 34585049 10.1002/ags3.12462 PMC8452469

[24] Lai F C, Chen L, Tu Y R et al. Prevention of chylothorax complicating extensive esophageal resection by mass ligation of thoracic duct: a random control study. Ann Thorac Surg 2011; 91(6): 1770–4. 21536248 10.1016/j.athoracsur.2011.02.070

[25] Yoshida N, Nagai Y, Baba Y et al. Effect of resection of the thoracic duct and surrounding lymph nodes on short- and long-term and nutritional outcomes after esophagectomy for esophageal cancer. Ann Surg Oncol 2019; 26(6): 1893–900. 30863941 10.1245/s10434-019-07304-z

[26] Oshikiri T, Takiguchi G, Miura S et al. Thoracic duct resection during esophagectomy does not contribute to improved prognosis in esophageal squamous cell carcinoma: a propensity score matched-cohort study. Ann Surg Oncol 2019; 26(12): 4053–61. 31313045 10.1245/s10434-019-07627-x

[27] Fujisawa K, Ohkura Y, Ueno M et al. Nutritional outcomes of thoracic duct resection for radical esophagectomy by assessing body composition changes in one year: a single-center retrospective study. Ann Surg Oncol 2021; 28(13): 8414–25. 34085142 10.1245/s10434-021-10222-8

[28] Nishimura E, Matsuda S, Kawakubo H et al. The impact of thoracic duct resection on the long-term body composition of patients who underwent esophagectomy for esophageal cancer and survived without recurrence. Dis Esophagus In press. 10.1093/dote/doad002 PMC10473448 37465862

